# Simple rapid stabilization method through citric acid modification for magnetite nanoparticles

**DOI:** 10.1038/s41598-020-67869-8

**Published:** 2020-07-01

**Authors:** Mohammed Ali Dheyab, Azlan Abdul Aziz, Mahmood S. Jameel, Osama Abu Noqta, Pegah Moradi Khaniabadi, Baharak Mehrdel

**Affiliations:** 1 Nano-Biotechnology Research and Innovation (NanoBRI), Institute for Research in Molecular Medicine (INFORMM), Universiti Sains Malaysia, 11800 Pulau Pinang, Malaysia; 2 Nano-Optoelectronics Research and Technology Lab (NORLab), School of Physics, Universiti Sains Malaysia, 11800 Pulau Pinang, Malaysia

**Keywords:** Materials science, Optics and photonics

## Abstract

A highly stable and magnetized citric acid (CA)-functionalized iron oxide aqueous colloidal solution (Fe_3_O_4_@CA) was synthesized by using a simple and rapid method of one-step co-participation via a chemical reaction between Fe^3+^ and Fe^2+^ in a NaOH solution at 65 °C, followed by CA addition to functionalize the Fe_3_O_4_ surface in 25 min. The NPs were synthesized at lower temperatures and shortened time compared with conventional methods. Surface functionalization is highly suggested because bare Fe_3_O_4_ nanoparticles (Fe_3_O_4_ NPs) are frequently deficient due to their low stability and hydrophilicity. Hence, 19 nm-sized Fe_3_O_4_ NPs coated with CA (Fe_3_O_4_@CA) were synthesized, and their microstructure, morphology, and magnetic properties were characterized using X-ray diffraction, transmission electron microscopy, Zeta potential, Fourier transform infrared spectroscopy, and vibrating sample magnetometer. CA successfully modified the Fe_3_O_4_ surface to obtain a stabilized (homogeneous and well dispersed) aqueous colloidal solution. The Zeta potential value of the as-prepared Fe_3_O_4_@CA increases from − 31 to − 45 mV. These CA-functionalized NPs with high magnetic saturation (54.8 emu/g) show promising biomedical applications.

## Introduction

Fe_3_O_4_ NPs with a grain size of smaller than 20 nm display superparamagnetic behavior at high temperatures but exhibit no coercivity and remanence at room temperature^1–4^. These particles are extensively utilized for several biomedical and in vivo applications^5–9^. Fe_3_O_4_ NPs, a well-known ferrofluid, has been expansively analyzed, particularly their colloidal dispersion and many potential biomedical applications. The surface of magnetite particles is modified by different coating agents, including protein^10^, methoxypoly (ethylene glycol)^11^, dextran^12^, chitosan^13^, and silica coating^14^, to enhance their performance. Controlling the sizes and dispersion of NPs in preferred solvents is technologically challenging due to difficulties faced in their fabrication and handling for biomedical applications, including their clustering/aggregation, homogeneity, hydrophilicity, and biocompatibility^15,16^. The high surface energies of NPs are attributed to their large surface to volume ratio. NPs tend to aggregate to minimize total surface energy, which exceeds 0.1 N/m for metal oxide surfaces^17^.

Proper functionalization of NP surface and solvent selection are critical to attain adequate repelling interactions between the NPs to inhibit agglomeration/accretion and improve the thermodynamic stability of the colloidal solution. The surface of Fe_3_O_4_ dispersed in aqueous media via citric acid adsorption can be functionalized by utilizing the coordination of one or two carboxylate functionalities of the citric acid depending on the steric necessity and curvature of the surface^18^. Carboxylates significantly affect the development of Fe_3_O_4_ NPs and their magnetic characteristics. Surface modification of aqueous magnetic NPs by using heavy chain fatty acid or thiol is one of the methods to increase the stability of NP suspension^19^. Co-precipitation is typically used to synthesize water-stable Fe_3_O_4_ NPs and considered as the simplest, most cost-effective technique requiring the lowest temperature^20^. However, its main drawbacks are the agglomeration, broad size distribution, poor Zeta potential values of NPs. Fe_3_O_4_ NPs also lack good colloidal stability and have inadequate repulsive forces to prevent agglomeration. The poor colloidal stability and broad size distribution can be attributed to the reaction time and temperature during co-precipitation. To overcome these problems, the Fe_3_O_4_ NPs must be stabilized and their size distribution must be reduced by modifying their surfaces with biocompatible materials, in addition to controlling the synthesis procedures. Nevertheless, most of aqueous stabilized Fe_3_O_4_ NPs are achieved either at high temperature^21–23^ or long reaction time^24–26^. For example, Elham et al.^27^ and Arefi et al.^28^ synthesized citric acid (CA)-stabilized Fe_3_O_4_ NPs through two-step co-precipitation that is laborious and time consuming. In addition, Singh et al.^29^ synthesized CA-coated Fe_3_O_4_ NPs through co-precipitation, and the transmission electron microscopy (TEM) results indicated that the NPs have agglomerated and are non-uniform in shape.

To the extent of our knowledge, the stability of Fe_3_O_4_@CA NPs has not been reported. Therefore, this study aims (1) to synthesize a highly stable and magnetized Fe_3_O_4_@CA aqueous colloidal solution by employing a one-step, fast, and straightforward route (with shortened time and lower temperature than conventional methods) and systematically controlling and manipulating the flow of the reaction procedure and (2) to develop surface functional groups on magnetic NP derivatization through a one-step process.

## Materials and methods

### Materials

Ferric chloride (FeCl_3_·6H_2_O, 99%), ferrous chloride (FeCl_2_·4H_2_O, 99%), and sodium hydroxide (NaOH) were acquired from Sigma–Aldrich, and citric acid (CA) were purchased from Merck.

### Preparation Fe_3_O_4_@CA

**Figure 1:**
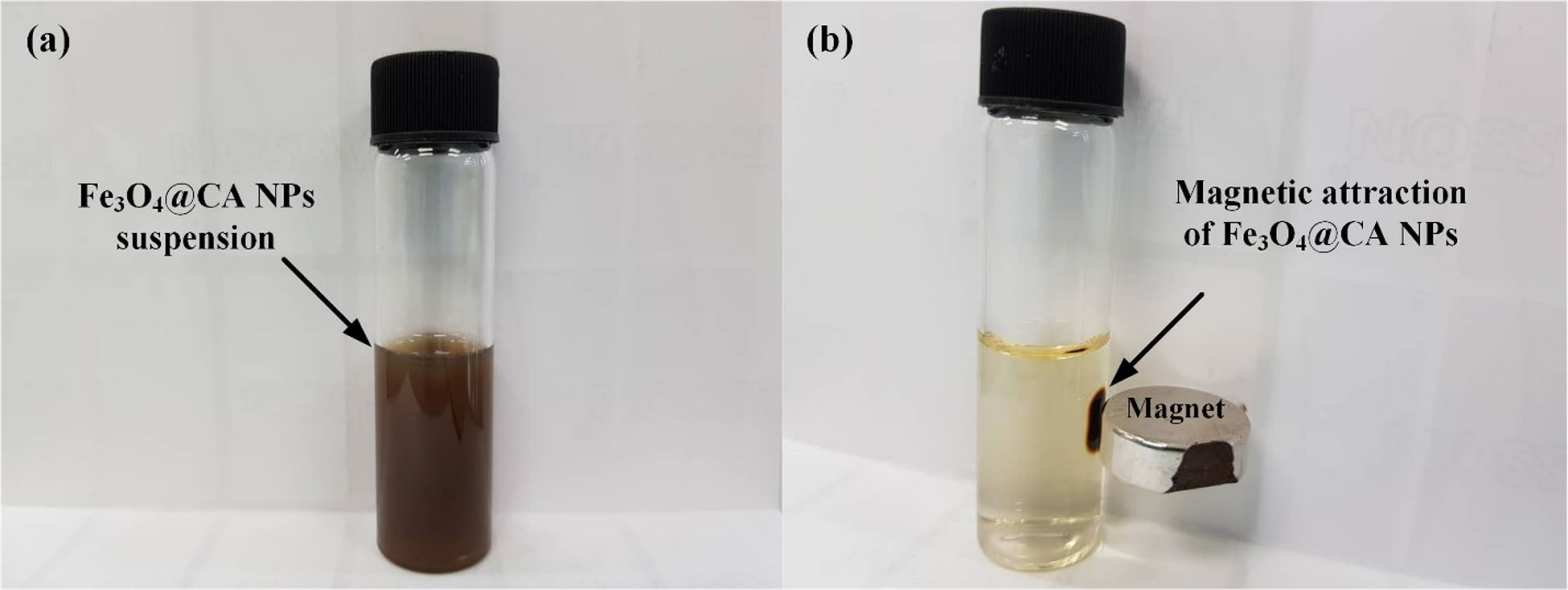
Magnetic attraction of Fe_3_O_4_@CA NPs: (**a**) Fe_3_O_4_@CANPs in solution state and (**b**) magnetic attraction of Fe_3_O_4_@CA NPs toward a magnet.

Fe_3_O_4_ NPs were synthesized through the co-precipitation of ferrous (Fe^2+^) and ferric (Fe^3+^) with sodium hydroxide (NaOH). FeCl_2_.4H_2_O (2.5 g) and FeCl_3_.6H_2_O (4.0 g) were dissolved in 180 mL of distilled water under nitrogen gas. Following the complete dissolution of the mixture at room temperature, 50 mL of sodium hydroxide was drop-wise added to the reaction mixture, which was mechanically stirred at 650 rpm and kept for 10 min at 65 °C under continuous vigorous stirring. For the prevention of Fe_3_O_4_ NP agglomeration, 150 mL of CA was added to the reaction mixture, which was then stirred for 10 min (65 °C). Fe_3_O_4_@CA NPs were collected through a permanent magnet and thoroughly rinsed four times with distilled water to eliminate unreactive or inert impurities. Finally, the Fe_3_O_4_@CA NPs were redispersed in the distilled water after sonication for 5 min, and the resulting suspension (Fe_3_O_4_@CA) responded to an external magnetic field as shown in Fig. 1.

### Characterization of Fe_3_O_4_@CA

X-ray diffraction (XRD) patterns were obtained using an X-ray diffractometer (PANalytical X’pert PRO MRD PW 3040) with CuKa (λ = 1.54050 Å). The size of Fe_3_O_4_ NPs and Fe_3_O_4_@Au CSNPs were obtained by transmission electron microscopic (TEM) using a Zeiss Libra 120 at 100 kV. Particle size distribution was measured using ImageJ software. The stability (Zeta potential) of Fe_3_O_4_@CA NPs was described using a dynamic light scattering (DLS) instrument (ZETASIZER Nanoseries Model ZEN 3600, Malvern Instruments). The surface functional groups of Fe_3_O_4_@CA NPs were determined by Fourier transform infrared spectroscopy (PERKIN ELMER System 2000 FT-IR). Magnetic properties were evaluated using vibrating sample magnetometer (VSM, DMS MODEL 8810).

## Results and discussion

**Figure 2:**
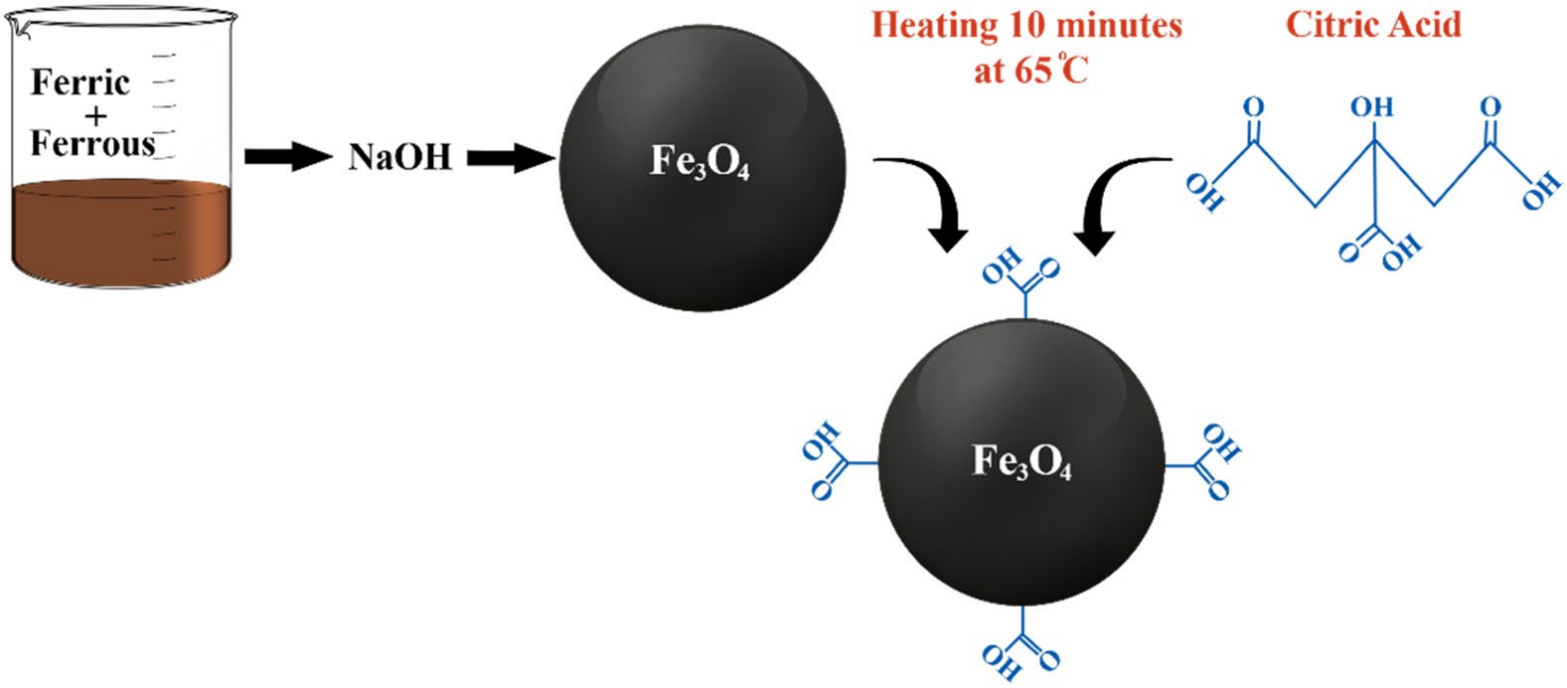
Steps for CA functionalization of Fe_3_O_4_ NP surface in 25 min.

Fe_3_O_4_ NP surfaces were functionalized via CA adsorption, which occurs by coordinating one or two of the carboxylate functionalities depending on the need for steric repelling to stabilize the ferrofluids and the curvature or morphology of the surface. Nonetheless, a minimum of one carboxylic acid group is exposed to the solvent, thus accounting for the surface charging. The presence of a carboxylic group surface ligand offers the possibility of developing bonds with proteins, fluorescent dyes, and hormone linkers to facilitate precise targeting in biological systems. The one-step modification of the superparamagnetic Fe_3_O_4_ NP surface is presented in Fig. 2, and the as-prepared Fe_3_O_4_ NPs were subsequently stabilized with CA to prevent agglomeration.

**Figure 3:**
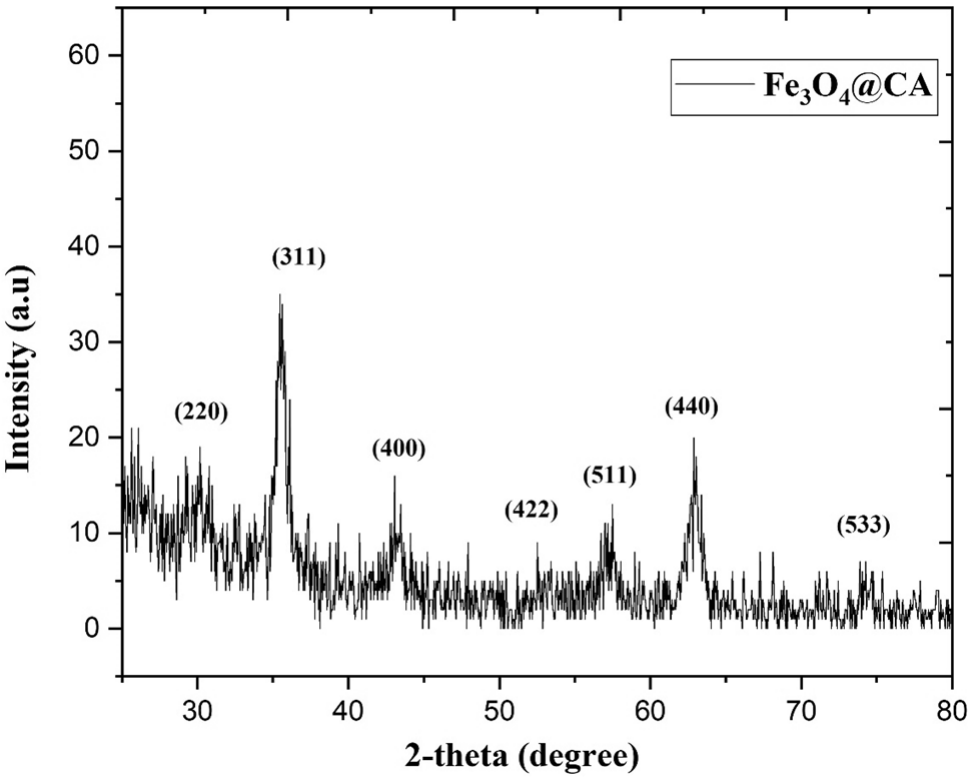
XRD spectra of the CA-functionalized Fe_3_O_4_ NPs showing the composition and crystal structure of Fe_3_O_4_.

The XRD spectra (Fig. 3) of Fe_3_O_4_ NPs confirmed their cubic spinel structure with high crystallinity. These diffraction peaks are narrow and well defined, indicating the high crystallinity of the sample. The positions and intensities of the diffraction peaks for NPs are consistent those of Fe_3_O_4_ (Ref. Code 01-075-0033). The synthesis method for Fe_3_O_4_ particles via the co-precipitation of Fe^2+^ and Fe^3+^ in an aqueous base solution is a relatively established and extensively utilized procedure^25,30^. The XRD results are consistent with the possible constituents of Fe_3_O_4_ particles. The diffraction peaks denote the crystallinity of Fe_3_O_4_ NPs as spinel cubic lattice type. Nevertheless, further oxidation of the Fe_2_O_3_ phase was not verified by the XRD data due to the similarity between lattice type and constant^31^. According to a previous study, the CA coating for Fe_3_O_4_ NPs does not result in the phase change in the XRD spectra of bare Fe_3_O_4_^29^.

**Figure 4:**
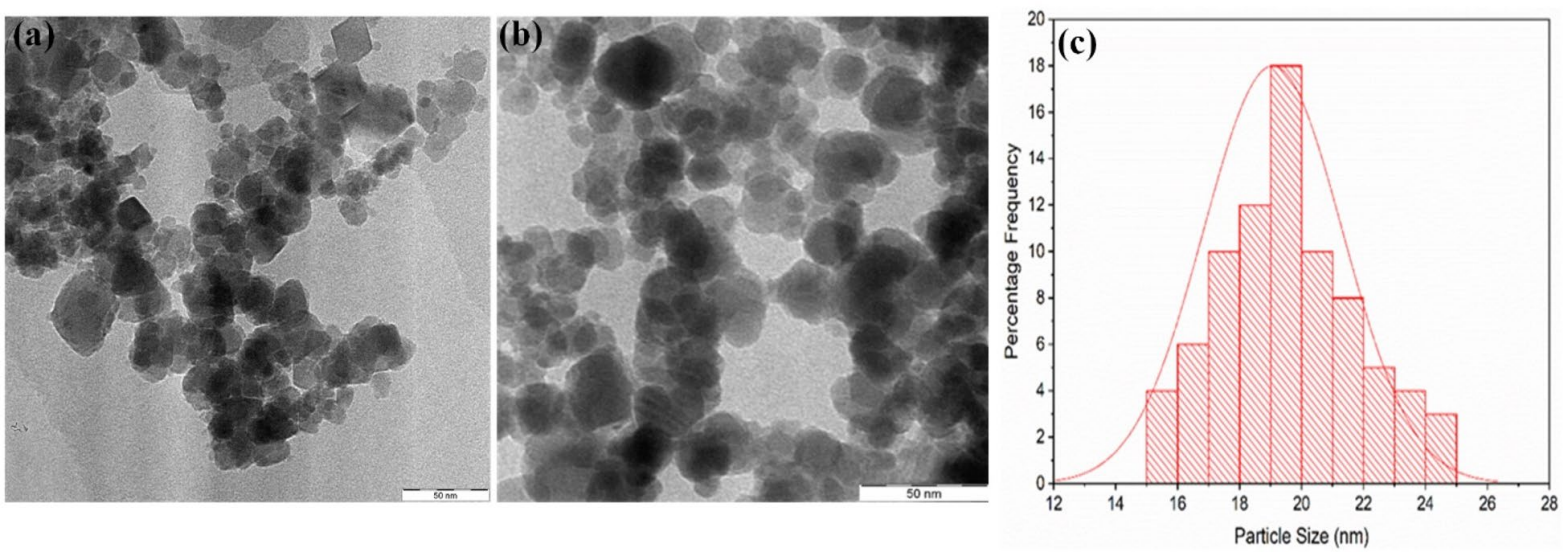
TEM micrographs of (**a**) bare Fe_3_O_4_, (**b**) Fe_3_O_4_@CA, and (**c**) the corresponding size distribution of Fe_3_O_4_@CA.

The TEM image and size distribution of the as-synthesized Fe_3_O_4_ and Fe_3_O_4_@CA NPs are presented in (Fig. 4). Figure 4a shows the TEM image of Fe_3_O_4_ prior to CA modification. A slightly important change in Fe_3_O_4_ agglomeration was induced by CA. From the histogram in Fig. 4c, the average size of the monodispersed Fe_3_O_4_@CA NPs is approximately 19 nm. The Fe_3_O_4_@CA NPs are spherical in shape with a narrow size distribution after CA modification, particularly at stable synthesis conditions. The TEM images of the CA-functionalized superparamagnetic Fe_3_O_4_ NPs show semi-spherical shaped particles and monodispersion. Previous studies used co-precipitation to synthesize CA-coated Fe_3_O_4_ and produced agglomerated NPs with average sizes 51^28^, 50^32^, 25,^33^ and 22 nm^34^, which might be due to the high reaction temperature^35^.

**Figure 5:**
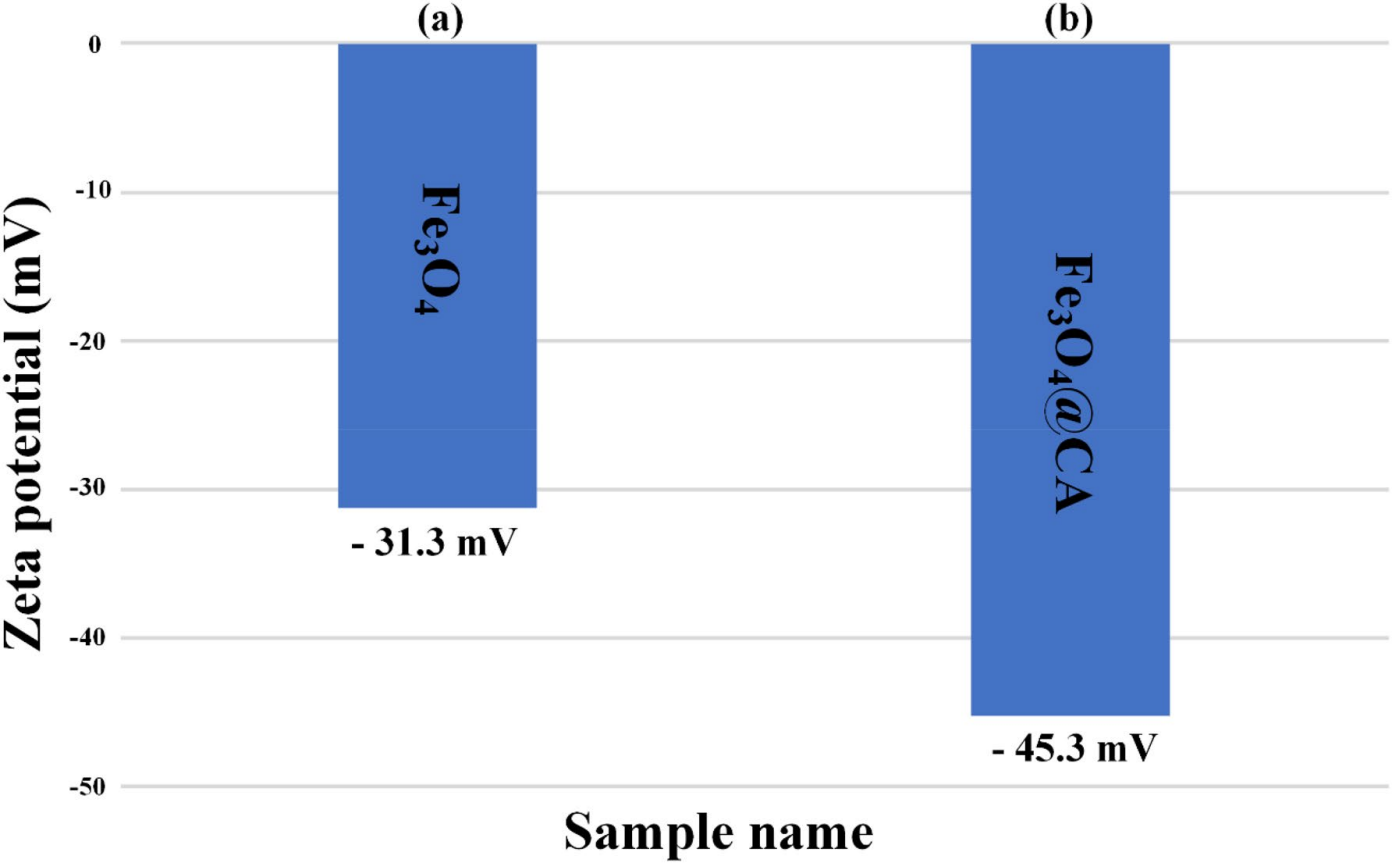
Stability measurement of as-synthesized nanoparticles using Zeta potential for (**a**) bare Fe_3_O_4_ and (**b**) Fe_3_O_4_@CA.

Figure 5 presents the stability of Fe_3_O_4_ after CA modification to show the role of CA on the stability of Fe_3_O_4_ NPs. The Zeta potential magnitude of Fe_3_O_4_ NPs was measured immediately after the synthesis of the particles, followed by CA injection on the colloidal Fe_3_O_4_ NPs. Dispersion stability can be defined in relation to the Zeta potential value (mV): 0 to ± 5 can cause the rapid agglomeration and precipitation of NP suspension, ± 10 to ± 30 is responsible for the threshold of delicate dispersion, ± 30 to ± 40 denotes the moderate stability of colloidal NPs^36–39^, and ± 40 to ± 60 indicates the excellent stability of NP suspension referred to as a high charge on their surface^40^. The measured results indicate that Zeta potential improves from − 31.3 to − 45.3 mV (Fig. 5a, b), which is higher than the reported values^41–43^ and is attributed to the three carboxylate groups of citrate that dissociate and strongly bind with Fe_3_O_4_ NP surface^18^. In addition, the negative charge of the Zeta potential is due to the electrostatic stabilization provided by the strong adsorption of citrate ions on NP surface. The Zeta potential of CA-coated Fe_3_O_4_ has not been reported. The increase in the measured Zeta potential revealed that CA is absorbed onto the Fe_3_O_4_ NP surface, thus resulting in a highly negative surface charge. The presence of carboxylate group is confirmed by monitoring the Zeta potential for Fe_3_O_4_ NPs. However, this study characterized and analyzed only the results for a moderately polydispersed sample.

**Figure 6:**
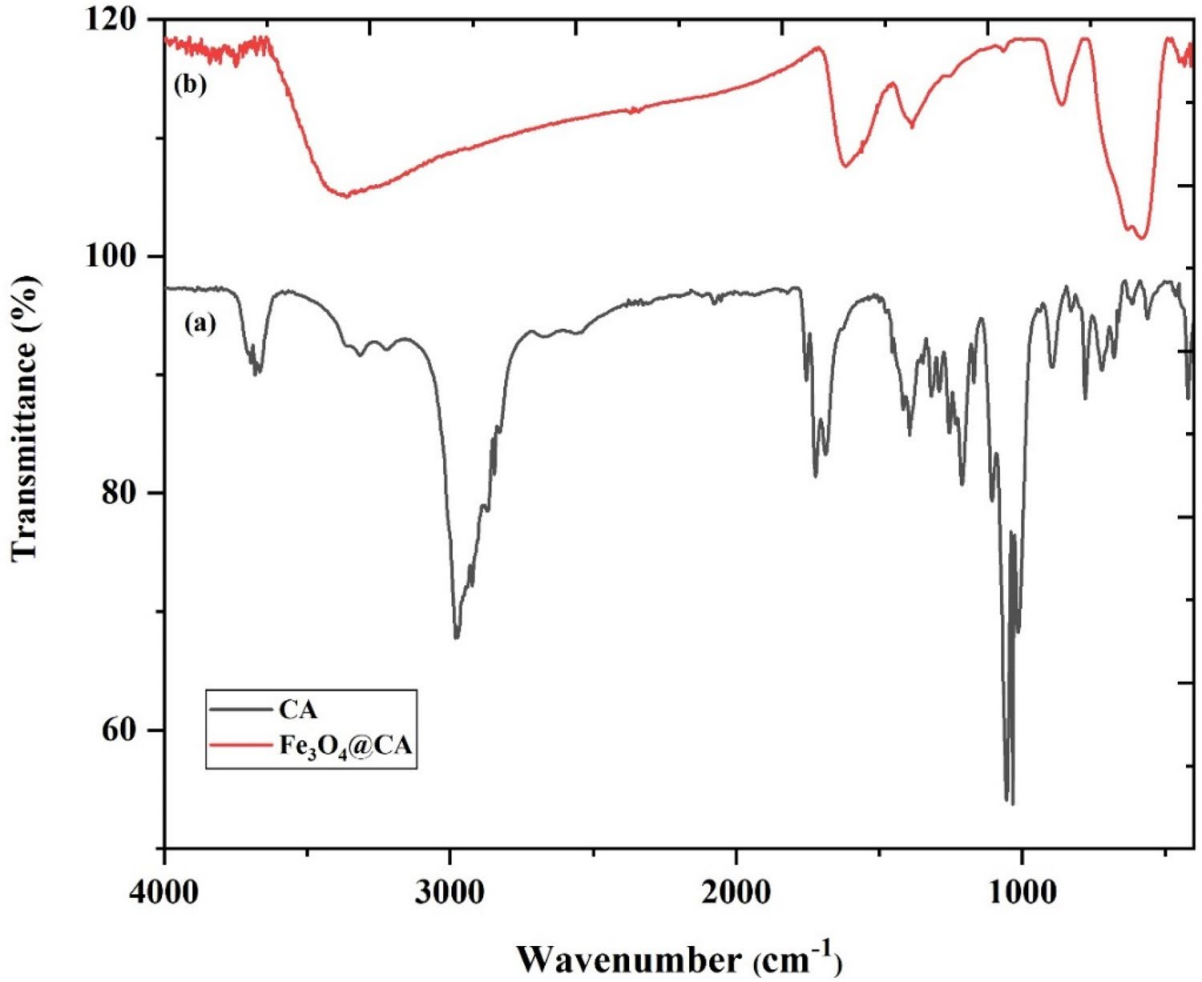
FTIR spectra of (**a**) bare CA and (**b**) CA conjugated on the surface of Fe_3_O_4_ NPs.

The Fourier transform-infrared spectroscopy (FT-IR) spectrum of bare Fe_3_O_4_ has been reported^28,29^. This study aimed to prove the presence of CA on the Fe_3_O_4_ surface. FT-IR spectra of CA and Fe_3_O_4_@CA NPs are illustrated in Fig. 6a and b. The spectrum peak was assigned to the CA-coated Fe_3_O_4_ NPs. The broad band spectrum at 3,384 cm^−1^ can be referred to as the OH band groups and to the traces of molecular water. The 1722 cm^−1^ spectrum peak of CA is due to the symmetric C=O stretching from the COOH group. This peak display shifts to a lower wavelength at approximately 1615 cm^−1^ for the carboxylic group (R-OOH) of the Fe_3_O_4_@CA. The peak at 1615 cm^−1^ determines the binding of CA radical on the surface of Fe_3_O_4_ NPs through the chemisorption of carboxylate citrate ions^28,29^. The peak at 1,384 cm^−1^ can be ascribed to the asymmetric stretching of C–O from the carboxylic group. The intense peak observed at the IR range at approximately 578 cm^−1^ in Fe_3_O_4_@CA could be assigned to the Fe–O stretching vibrational mode of Fe_3_O_4_^44^. Hence, CA binds to the Fe_3_O_4_ surface through carboxylate.

**Figure 7:**
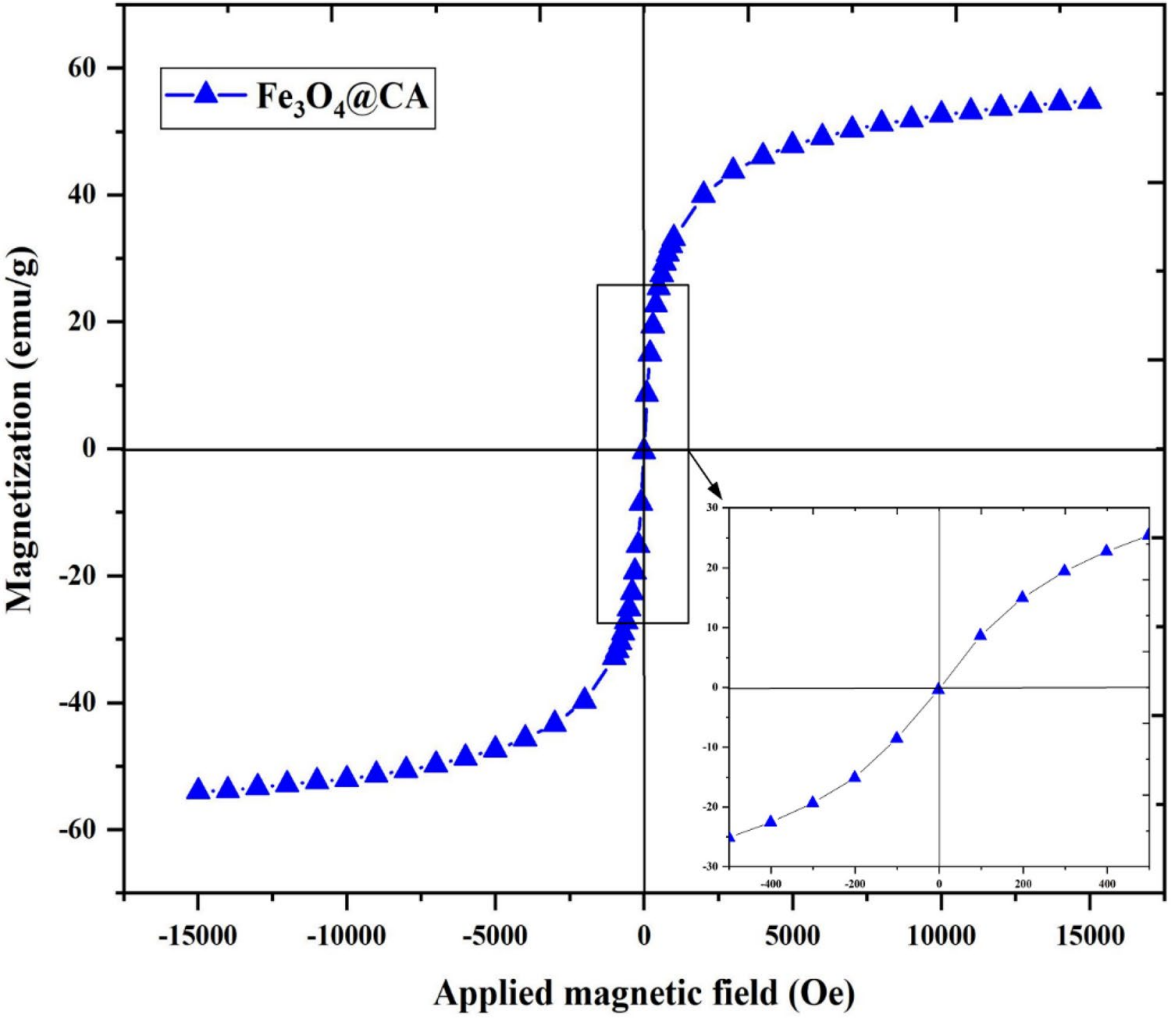
Magnetization curve of superparamagnetic (no coercivity or remanence) Fe_3_O_4_@CA at room temperature.

The magnetic properties of Fe_3_O_4_@CA were determined by VSM analysis at room temperature. The magnetization saturation (emu/g) as a function of the applied magnetic field (Oe) is illustrated in Fig. 7. The magnetization curve shows that the Fe_3_O_4_@CA NPs exhibit a superparamagnetic behavior and magnetic saturation (Ms) of approximately 54.8 emu/g, which is higher than that in previous studies^25,33^ (Table 1) possibly due to the low Fe oxidation state. No hysteresis was observed, and the behavior was completely reversible at 300 K. Neither coercivity nor remanence was observed. Arefi et al.^28^. reported that the Ms of bare Fe_3_O_4_ is reduced after being coated with CA. Alonso et al.^23^ synthesized Fe_3_O_4_ NPs with high crystallinity of approximately 35 nm and high Ms of 65 emu/g by using thermal decomposition. The high Ms value is attributed to the large particle size of Fe_3_O_4_^45^. Therefore, the Ms of Fe_3_O_4_ NPs decreases with their reduced particle size due to the increase in surface spin disorder^46,47^. In this case, the size reduction to the nanoscale (below 20 nm) for spherical single-component Fe_3_O_4_ greatly influences the magnetic ordering of surface spins, namely, a high degree of disordered surface spins of Fe_3_O_4_ NPs. Spherical Fe_3_O_4_ NPs could develop surface spin disorder through energy minimization. The disordered surface spins are highly anisotropic, which is in line with the increase in their effective magnetic anisotropy.

**Table 1:** Comparison with previous studies on Fe_3_O_4_ synthesis.

No.	Method	Reaction Time (minutes)	Temperature °C	Saturation magnetization (emu/g)	Ref.
1	Co-precipitation	60	90	46	^33^
2	Co-precipitation	120	–	32.40	^25^
3	Co-precipitation	150	90	74	^27^
4	Co-precipitation	120	–	55	^28^
5	Co-precipitation	35	90	–	^29^
6	Present study	25	65	54.8	

For biomedical applications such as in hyperthermia and magnetic resonance imaging (MRI), NPs must have a uniform particle size, exhibit superparamagnetism, and possess high Ms. The as-synthesized Fe_3_O_4_@CA has a high magnetic response, which is preferable for biomedical applications^28^. Our method shows an advantage of having a simple and rapid route to synthesize highly stable (− 45.3 mV), monodispersed, and superparamagnetic Fe_3_O_4_@CA (19 nm) compared with conventional techniques.

## Conclusion

We developed a simple and rapid synthesis route for highly stable and superparamagnetic Fe_3_O_4_@CA through co-precipitation. The proposed method requires simple equipment and cheap materials such as a magnetic stirrer, and the processing time was 25 min at 65 °C. The NPs were achieved at lower temperature, simpler process, and shorter time compared with conventional methods. XRD, TEM, Zeta potential, FT-IR, and VSM were employed to characterize the microstructure and morphology of the synthesized NPs. The presence of carboxylate group is confirmed by FTIR analysis, and the Zeta potential for Fe_3_O_4_ particles was monitored. The Zeta potential value of as-prepared Fe_3_O_4_@CA increased from − 31.3 to − 45.3 mV. Finally, these NPs are important for several biomedical applications due to their small size, stability, and superparamagnetic behavior.

## Supplementary information

Supplementary file1 (PDF 301 kb)
