# Supplementary material for: Simple rapid stabilization method through citric acid modification for magnetite nanoparticles
Source: Sci Rep. 2020 Jul 1;10:10793. doi: 10.1038/s41598-020-67869-8 (PMC7330025; doi:10.1038/s41598-020-67869-8)
Supplement: Supplementary file 1 — Supplementary file1 (PDF 301 kb) [file 41598_2020_67869_MOESM1_ESM.pdf]

Supplementary information

Simple Rapid Stabilization Method through Citric Acid Modification for Magnetite Nanoparticles

Mohammed Ali Dheyab[1,2]*, Azlan Abdul Aziz[1,2*], Mahmood S Jameel[1,2], Osama Abu Noqta[1,2], Pegah Moradi Khaniabadi[1,2], Baharak Mehrdel[1,2]

[1]Nano-Biotechnology Research and Innovation (NanoBRI), Institute for Research in Molecular Medicine (INFORMM), Universiti Sains Malaysia, 11800, Pulau Pinang, Malaysia
[2]Nano-Optoelectronics Research and Technology Lab (NORLab), School of Physics, Universiti Sains Malaysia, 11800 Pulau Pinang, Malaysia.

*Corresponding author: mohammed@student.usm.my, lan@usm.my

*Correspondence and requests for materials should be addressed to M.A.D and A.A.A. (email: mohammed@student.usm.my, lan@usm.my; 0060136460997).

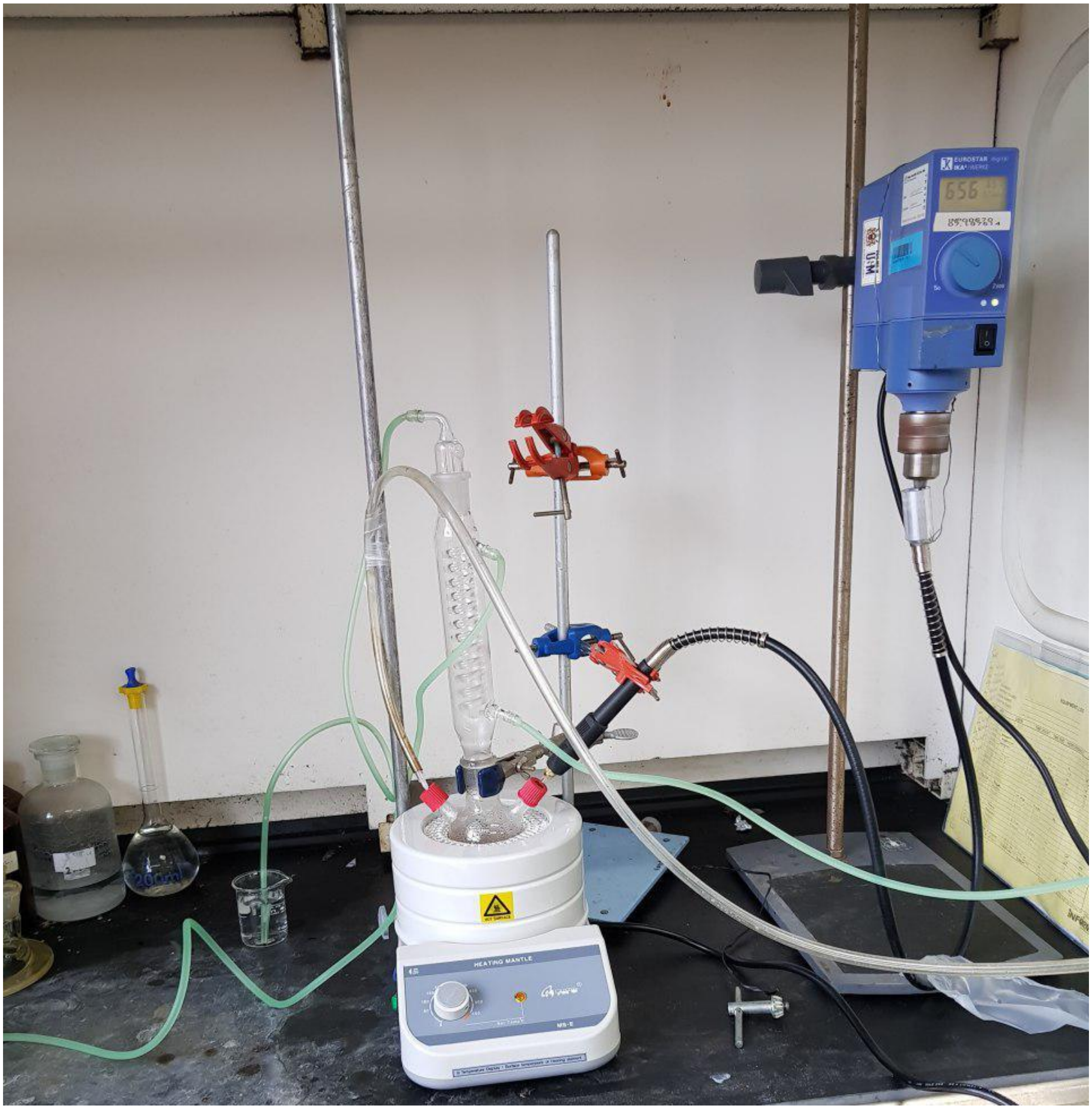

Supplementary Figure S1. These experimental setups and equipment were used to produce Fe_3O_4@CA nanoparticles.
